# Development of a novel and efficient cell culture flocculation process using a stimulus responsive polymer to streamline antibody purification processes

**DOI:** 10.1002/bit.24969

**Published:** 2013-06-29

**Authors:** Yun (Kenneth) Kang, James Hamzik, Michael Felo, Bo Qi, Julia Lee, Stanley Ng, Gregory Liebisch, Behnam Shanehsaz, Nripen Singh, Kris Persaud, Dale L Ludwig, Paul Balderes

**Affiliations:** 1 Bioprocess Sciences, ImClone Systems, a Wholly-Owned Subsidiary of Eli Lilly and Company450 East 29th Street, New York, New York, 10016; 2 Biopharm Process Solutions R&D, EMD MilliporeBillerica, Massachusetts; 3 Biomanufacturing Sciences Network, EMD MilliporeBillerica, Massachusetts; 4 Process Development, ImClone Systems, a Wholly-Owned Subsidiary of Eli Lilly and CompanyBranchburg, New Jersey

**Keywords:** flocculation, precipitation, stimulus responsive polymer, smart polymer, harvest, depth filtration, monoclonal antibody, purification, residual polymer, impurity removal, salt tolerant, hydrophobic interaction, HCP, aggregates, HMW, host DNA

## Abstract

Recent advances in mammalian cell culture processes have significantly increased product titers, but have also resulted in substantial increases in cell density and cellular debris as well as process and product related impurities. As such, with improvements in titer, corresponding improvements in downstream processing are essential. In this study we have developed an alternative antibody harvest process that incorporates flocculation using a novel stimulus responsive polymer, benzylated poly(allylamine), followed by depth filtration. As tested on multiple antibodies, this process demonstrates high process yield, improved clearance of cells and cell debris, and efficient reduction of aggregates, host cell proteins (HCP) and DNA. A wide operating window was established for this novel flocculation process through design of experiments condition screening and optimization. Residual levels of impurities in the Protein A eluate were achieved that potentially meet requirements of drug substance and thus alleviate the burden for further impurities removal in subsequent chromatography steps. In addition, efficient clearance of residual polymer was demonstrated using a fluorescence tagged polymer in the presence of a stimulus reagent. The mechanism of HCP and aggregates removal during flocculation was also explored. This novel and efficient process can be easily integrated into current mAb purification platforms, and may overcome downstream processing challenges. Biotechnol. Bioeng. 2013;110: 2928–2937. © 2013 Wiley Periodicals, Inc.

## Introduction

The first step of downstream operations in monoclonal antibody (mAb) manufacturing is the removal of cells and cellular debris from cell culture via centrifugation and depth filtration, prior to capture chromatography (Han et al., 2003; Kelley et al., 2009; Shukla et al., 2007). The separation of cells and cell debris can be facilitated by the addition of flocculants (Aunins and Wang, 1989; Han et al., 2003; Roush and Lu, 2008; Thommes and Etzel, 2007). Different flocculants and precipitants have been explored as components to improve clarification efficiency, process yield, and clearance of impurities during the primary mAb recovery step from mammalian cell culture (Hove et al., 2010; McNerney et al., 2011; Peram et al., 2010; Romero et al., 2010; Roush and Lu, 2008; Schirmer et al., 2010; Shan et al., 1996; Wang et al., 2009). Notably, positively charged flocculants, in particular polyamines (Peram et al., 2010), divalent cations (Romero et al., 2010; Shpritzer et al., 2006), chitosan (Riske et al., 2007), and polydiallyldimethylammonium chloride (PDADMAC) (McNerney et al., 2011; Zhao et al., 2012) have been shown to be successful in inducing flocculation as a result of interactions between the flocculant and the negatively charged surfaces of cells and cell debris. A key benefit of flocculation is a potential reduction in process related impurities such as HCP, host DNA, and viruses (Akeprathumchai et al., 1). However, purification performance in this step is culture dependent (Roush and Lu, 2008) and it would be challenging to develop this step as an alternative to downstream polishing chromatography steps. Further, due to their toxicity, flocculants must be removed from the final antibody product (Gagnon, 2012; Schirmer et al., 2010), possibly requiring the inclusion of an additional separation step and in-process monitoring or clearance studies of residual polymers.

In this study, a novel stimulus responsive polymer (Jaber et al., 2011), partially benzylated poly(allylamine), termed smart polymer E (SmP E) in this article, was investigated as a potential flocculant to address the mAb clarification and purification challenges discussed above. SmP E is a salt tolerant, cationic polymer with hydrophobic residues that undergoes a soluble to insoluble transition when exposed to multivalent anions such as phosphate ions and thus can be employed for flocculation and/or precipitation. The polymer response to the stimulus of multivalent anions is likely due to strong interactions between multivalent anions and the closely arranged amine repeat units present on the polymer, resulting in a collapse and aggregation of polymer chains which renders it insoluble. It is this unique property that allows its highly efficient binding to negatively charged impurities such as HCP and host DNA under typical cell culture conditions while enhancing the removal of cells and cell debris and controlling the level of residual polymer. Through design of experiments (DOE) condition screening and optimization of flocculation in hundreds of experimental runs, we developed an improved harvest process by flocculation followed by depth filtration. The novel process demonstrates high step recovery, improved clearance of cells and cell debris, as well as efficient reduction of process and product related impurities such as HCP, host DNA, and high molecular weight species (HMW) for four different mAbs. Improvement in filterability was observed during the depth filtration step using Clarisolve™ 40MS filters. This process achieved levels of residual impurities in the Protein A eluate that meet the requirements of drug substance and thus alleviates the purification burden in subsequent chromatography operations.

In addition, the efficient clearance of residual polymer was demonstrated using fluorescence tagged SmP E. The residual polymer level was reduced to <0.1 ppm in the subsequent Protein A chromatography. Therefore, no additional ion exchange chromatography is likely needed for polymer removal. The mechanism of impurity removal in this new harvest/clarification step was also explored. The developed process can be easily incorporated into current purification platforms, offering a solution in cases where mAbs demonstrate poor purification process performance. Methods described here for developing the SmP E flocculation process could also be applied to process development using other defined flocculants.

## Materials and Methods

### Polymers and Chemicals

Partially benzylated poly(allylamine) (PAAm-Bn) was obtained as a yellow glassy solid and was dissolved in 1 M acetic acid to make a stock solution of 10% (w/v in g/mL). We again have termed PAAm-Bn in this article the smart polymer, or SmP for abbreviation. Two different polymers, SmP E and C with a molecular weight of 15 and 150 kDa, respectively, were evaluated in this study. The fluorescence labeling procedure of SmP and Poly (ethyleneimine) (PEI [∼60 kDa], Sigma–Aldrich, St. Louis, MA) can be found in Supplementary section (S1). PDADMAC with an average molecular weight of 200–350 kDa was purchased from Sigma–Aldrich. All buffer chemicals were purchased from JT Baker (Phillipsburg, NJ). Both PDADMAC and PEI were used in the present study as flocculant controls.

## Cell Culture

The mAbs used for this study were fully human IgG1 produced in Chinese hamster ovary (CHO) cells grown in fed-batch mode using a serum-free medium in an overhead stirred bioreactor vessel (Bellco Biotechnology, Vineland, NJ). The cultures were harvested on day 15 with terminal cell viabilities of 0–40% and total densities of 4.0–6.0 × 10^6^ cells/mL with a typical mAb titer of 0.4–5.0 g/L. In addition, a bispecific antibody, Mab-I, was produced under similar cell culture conditions.

## SmP Flocculation of Cell Culture

First, a 10-mL cell culture containing Mab-A was treated with a SmP E or C at concentrations of 0–0.8% (w/v) in the absence or presence of 50 mM stimulus reagent, sodium phosphate. The flocculated cell culture was centrifuged at 1,000 × g for 1 min (Beckman Coulter, Brea, CA), followed by filtration using 0.22 µm Express® SHC filter (EMD Millipore, Billerica, MA). Centrate turbidity was measured using a Hach 2100 portable turbidimeter (Loveland, CO). The process yield in the flocculation/clarification step was calculated and filtrate quality was evaluated for turbidity, HCP, and host DNA.

The flocculation conditions were then optimized using SmP E with a full factorial DOE at a 5-mL culture scale using two cationic polymer flocculants, PEI and PDADMAC, as controls. The buffer pH condition was evaluated at 4-level (5, 6, 7, and 8), SmP dose at 6-level (0%, 0.05%, 0.1%, 0.2%, 0.4%, and 0.8%), and stimulus reagent (sodium phosphate) at 5-level (0, 10, 40, 70, and 100 mM). To facilitate the detection of residual polymers, boron-dipyrromethene (BODIPY®) labeled SmP and PEI were prepared and used in this study. After the polymer was added into the cell culture followed by the stimulus, the culture pH was adjusted to pre-defined pH conditions using 2 M Tris base solution. The cell culture was then clarified by centrifugation at 1,000 × g for 1 min followed by filtration using 0.22 µm Express SHC filter. The filtrate was collected as the product stream for further analysis. Four response parameters, turbidity, process yield, HCP and residual polymer, were determined for each experimental run. The response surfaces were further defined in a group of experiments at a 30-mL culture scale through a central composite design (pH 6.0–7.0; SmP E: 0.1–0.4%; stimulus: 10–40 mM) with eight center points. In addition, flocculation was evaluated at the center point conditions with untagged SmP E.

HMW reduction during the flocculation was determined in another group of experiments using the same central composite design at 1-mL working volume using a Protein A purified bispecific antibody, Mab-I. The protein preparation contained 10 mM sodium phosphate, 140 mM NaCl, pH 7.4 at 5 mg/mL with a starting HMW level of 11.1%. The flocculated solutions were clarified through 0.2 µm filter (Nalgene, Rochester, NY) and tested for HMW contents using SE-HPLC. All experimental designs and data processing in this study were performed using JMP version 8.0 software (Cary, NC).

## Clarification/Filtration of Flocculated Cell Culture

Flocculation with fluorescence tagged and untagged SmP E was next performed under both optimized and worst-case scenario conditions at the 2-L culture scale. The treated cell cultures were clarified using the Clarisolve™ 40MS or 60HX depth filter (EMD Millipore). The untreated cell culture was clarified using Millistak+® D0HC and X0HC depth filters (EMD Millipore) in series at a 1:1 filter area ratio. The capacities of the pre-wetted depth filters with an area of 23 cm^2^ were evaluated to a final pressure drop of 20 psi. For experiments in which a pressure drop of 20 psi was not reached, the achieved throughputs and related pressure drops were reported. The sterilizing grade filter capacities for depth filter pools were determined at a constant pressure of 10 psi using Express SHF and SHC filters using Optiscale® 25 devices (3.5 cm^2^, EMD Millipore).

## Protein A Chromatography

MabSelect SuRe™ Protein A (GEHC, Piscataway, NJ) was used to purify the antibody present in the harvested cell culture fluid after SmP E treatment using an AKTA Explorer under the control of UNICORN 5.0 (GEHC) as described previously (Kang et al., 2012).

## Analytical Techniques

The in-process samples and purified proteins were analyzed for product concentration, purity, and residual impurities as described previously (Kang et al., 2012). Briefly, antibody concentrations in cell cultures were determined by Octet Protein A titer assay (Pall Life Sciences, Port Washington, NY). Protein A purified antibodies were quantified through the absorbance at 280 nm, using a Nanodrop system (Thermo Scientific, Wilmington, DE). Size exclusion high performance liquid chromatography (SE-HPLC) was used to monitor the size heterogeneity of mAbs under native conditions on an Agilent HPLC system using Chemstation as the controlling software (Santa Clara, CA). A TSK-Gel G3000SW_XL_ column (Tosoh Bioscience, Montgomeryville, PA) was utilized to separate HMW impurities, monomers and fragments. The flocculation process yield was calculated as follows:


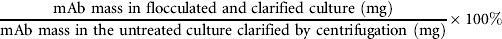


Similarly, the process yield in Protein A step was calculated using the following formula.


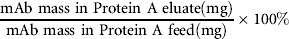


A CHO host cell protein kit (Cygnus, Southport, NC) was used to determine the residual HCP level in in-process samples and purified mAbs during the flocculation screening and optimization stages of experiments according to the manufacture s protocol. The HCP level in later stages was measured using the ELISA developed at ImClone with a quantification limit of 6.25 ng/mL. HCP results were normalized to the in-house CHO HCP standard. The leached MabSelect SuRe Protein A ligand in antibodies was determined using Repligen s Protein A ELISA kit (Waltham, MA) with a detection limit of 0.1 ng/mL according to the manufacture s protocol. Residual CHO DNA in antibodies was measured by quantitative PCR (qPCR) developed at ImClone using in-house CHO DNA standards. The quantification limit of the assay was 0.1 pg/mL.

Residual polymers were quantified by determining the fluorescence intensity from samples treated with the BODIPY tagged polymers using a SpectraMax M5^e^ microplate reader (Molecular Devices, Sunnyvale, CA). The excitation and emission maxima of the BODIPY tagged polymers were determined to be 508 and 550, respectively. The limit of quantification for this assay was 0.1 ppm.

## Results and Discussion

### Initial Screening Study

We first performed a flocculation screening study using two different polymers, SmP E and C, to identify which polymer and its concentration range to use in subsequent studies. Both SmP E and C in the dose range of 0.1–0.4% effectively reduced the turbidity in the product stream. Acceptable product recovery was achieved for all flocculation conditions tested. The SmP E or C treated cultures showed a high level of DNA clearance (>5 log reduction value, or LRV) while HCP levels were reduced by 30–70%, with slightly better performance observed using SmP E. As a result, SmP E was selected to use in follow-up studies. Since near complete clearance of DNA was observed across all the flocculating conditions, it was excluded from testing in the subsequent condition optimization. In the presence of 50 mM sodium phosphate, the level of residual SmP E detected in the clarified cell culture was <20 ppm. To the contrary, in the absence of 50 mM sodium phosphate, an elevated level of residual SmP E was observed when the polymer dose used for flocculation was ≥0.4%, suggesting the presence of excess free SmP and the need for a stimulus reagent to minimize residual SmP in the product stream.

## Flocculation Condition Optimization

**Figure 1:**
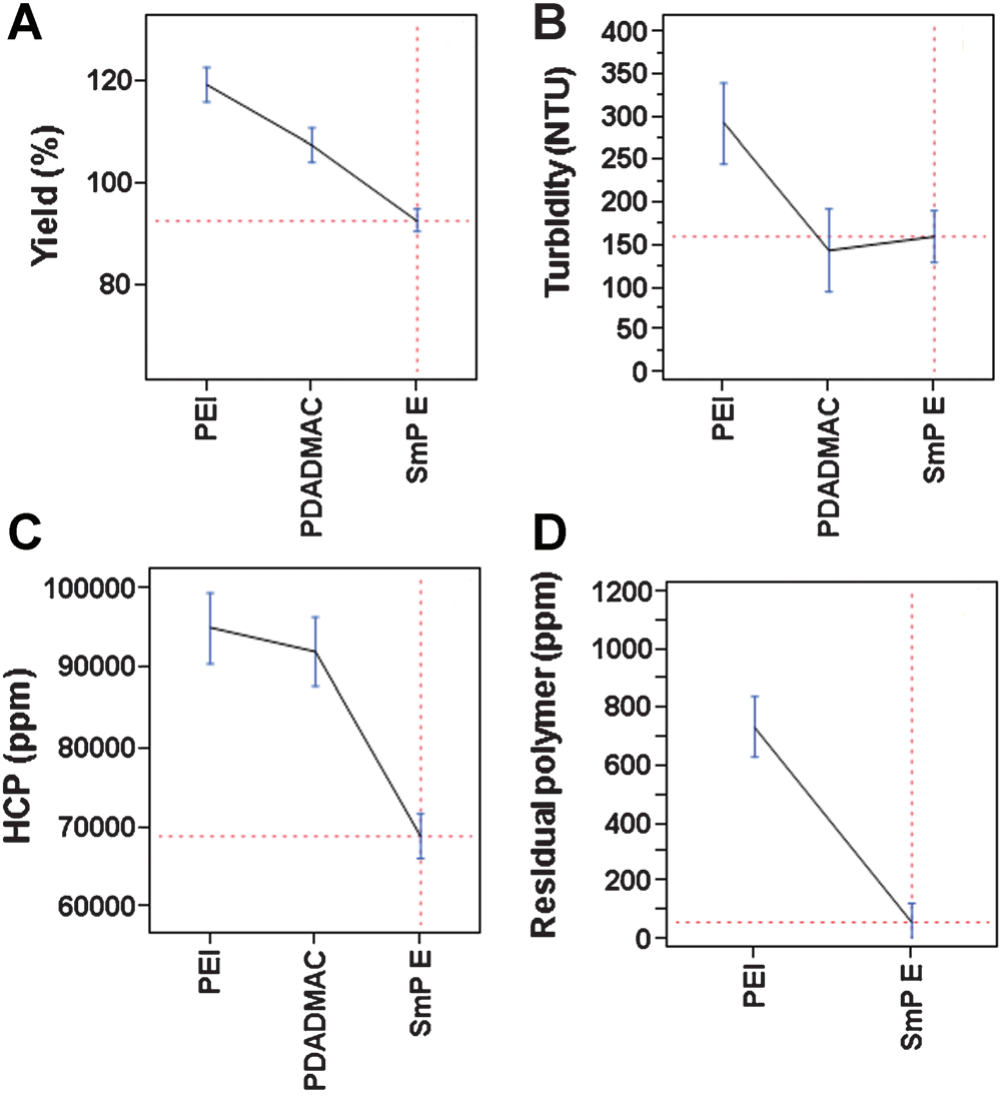
Cell culture flocculation process performance comparison of three polymers (SmP E, PDADMAC, and PEI) in terms of process yield (A), turbidity (B), HCP (C), and residual polymer (D). The same concentration ranging from 0% to 0.8% was used for the three polymers in the study.

We first tested cell culture preparations containing Mab-T in order to evaluate SmP E flocculation, with PDADMAC and PEI used as flocculant controls (described in the Materials and Methods Section). Challenges existed with the current purification process for Mab-T, so it was considered a worst-case scenario for these studies, in terms of residual HCP level in the Pro A eluate. The comparison of process performance using SmP E, PDADMAC, and PEI is shown in Figure 1 in terms of process yield, filtrate turbidity, HCP and residual polymer. An acceptable process yield was achieved in all experimental runs no matter which polymer was used. The unusually high level of process yield observed following PDADMAC or PEI flocculation may be due to interference from high levels of residual polymers in these samples impacting the reliability of the Octet titer assay. All polymers efficiently removed cells and cellular debris with slightly better performance observed using SmP E and PDADMAC (Fig. 1B). The residual HCP in SmP E flocculated cultures was significantly lower than that present in cell culture samples treated with PDADMAC or PEI. The robust HCP removal observed with SmP E may be due to its salt tolerant and hydrophobic nature (Fig. 1C). Consistently lower residual polymer was detected in SmP E treated cultures than observed in PEI treated cultures. Residual PDADMAC was not measured due to inherent challenges in fluorescence labeling of PDADMAC.

The significance of three operating parameters, pH, polymer dose, and stimulus concentration was assessed using a standard least squares model (Table 1). Both pH and polymer dose are considered significant flocculation parameters in terms of *p*-value or estimate for all evaluated response parameters. In contrast, addition of stimulus is important only with respect to turbidity and residual SmP E in the product stream.

**Table I:** Analysis of operating parameters in Mab-T/SmP E flocculation process using a standard least squares model

Operating factors\Responses	pH		Polymer dose (%)		Stimulus (mM)	
	*P*-value	Estimate	*P*-value	Estimate	*P*-value	Estimate
Yield (%)	<0.0001	−2.7	<0.0001	−21	0.1466	−0.02
Turbidity (NTU)	<0.0001	49	0.2625	−53	0.0003	1.3
Residual SmP (ppm)	0.003	−58	<0.0001	350	0.0337	−1
HCP (ppm)	<0.0001	10,146	<0.0001	−35711	0.3929	30

**Figure 2:**
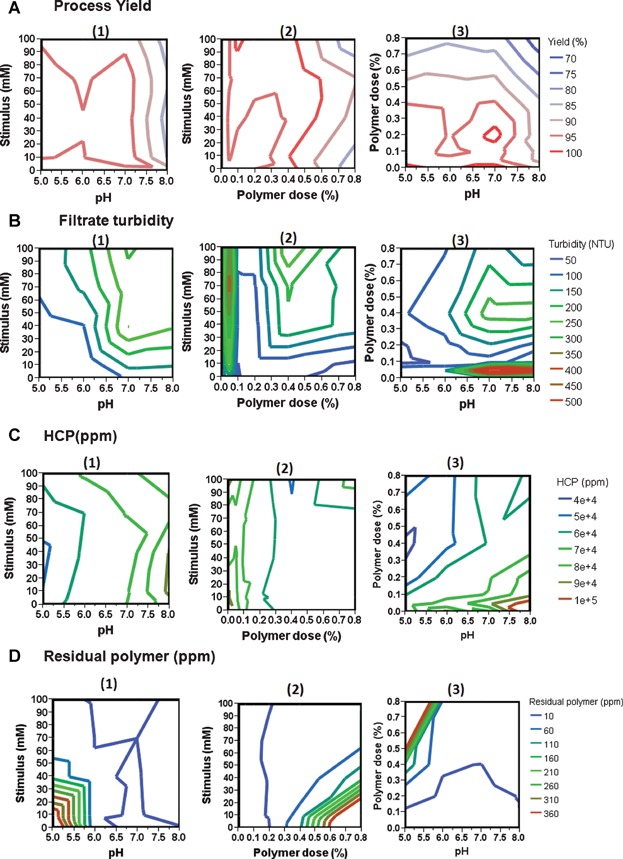
Contour plots from the operating condition optimization study of the Mab-T/SmP E flocculation process using a full factorial design. The effect of three input parameters (pH, polymer dose, stimulus concentration) was evaluated for four response parameters: (A) process yield; (B) turbidity; (C) HCP; (D) residual polymer. Starting HCP level was 105826.8 ppm.

The results of 120 experimental runs using SmP E were further summarized in Figure 2. Effects of polymer dose and operating pH on process yield were clearly demonstrated in Figure 2A(1–3). Greater than 90% process yield was achieved in all experimental runs except for runs with pH >7.5 and/or polymer dose >0.6%. The impact of stimulus on process yield was not significant.

The turbidity contour plot in Figure 2B (1) shows that decreasing flocculation pH from 8.0 to 5.0 progressively reduced the product stream turbidity, particularly when pH was <6.0. The strong impact of polymer dose on the turbidity is shown in Figures 2B(2) and (3). In some cases, increasing stimulus concentration from 0 to 100 mM slightly increased the product stream turbidity.

Similarly, the HCP contour plot in Figure 2C(1) indicated that decreasing flocculation pH from 8.0 to 5.0 progressively reduced the HCP level in the product streams, consistent with acid precipitation as reported previously (Romero et al., 2010). The strong impact of polymer dose on the HCP is shown in Figure 2C(2) and (3). Relatively high HCP level was observed when <0.1% of SmP E was added into the culture. A synergistic effect of low pH precipitation and SmP E flocculation on HCP reduction was also observed (Fig. 2C(3)). As expected, variation of stimulus concentration had limited effect on HCP clearance.

The results obtained for residual SmP E were appreciably different (Fig. 2D). A decrease in pH to <6.0 correlated with a significantly higher level of residual polymer (Fig. 2D(1)). A similar trend was also observed when the stimulus concentration was reduced to <40 mM. As expected, polymer dose had considerable impact on levels of observed residual polymer (Fig. 2D(2–3)).

In summary, operating ranges of pH (6–7), polymer dose (0.1–0.4%) and stimulus concentration (10–80 mM) in the flocculation process were identified as favorable conditions for the desired output and served as the basis for the experimental design in the next stage.

## Robustness Test of Flocculation Conditions

**Figure 3:**
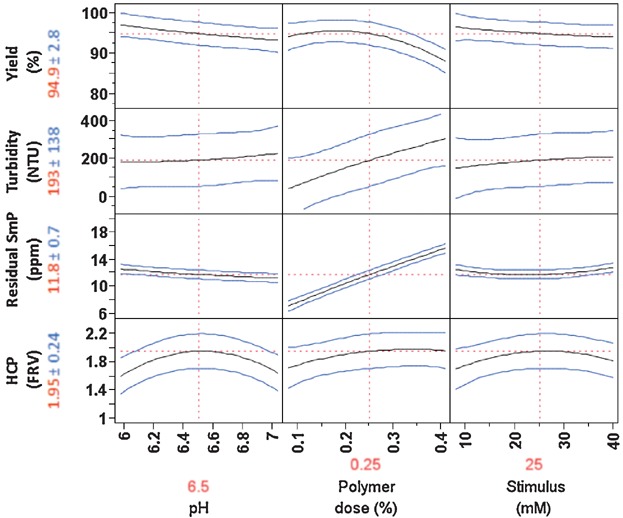
Prediction profiles for Mab-T/SmP E flocculation process robustness study using a central composite design. Process yield, turbidity, residual SmP E, and HCP fold reduction value (FRV) were analyzed as a function of pH, polymer dose, and stimulus concentration. The blue lines represent the confidence intervals of prediction profiles.

The process conditions developed in the previous section were further tested for their robustness through 12 additional flocculation runs using a central composite design (pH 6.0–7.0, polymer dose: 0.1–0.4% and stimulus concentration: 10–40 mM). The low stimulus concentration range was chosen in this stage to minimize the dilution factor during the flocculation process. The flocculation response surfaces of process yield, as well as HCP, turbidity and residual polymer in the product streams were defined based on these runs. The robustness of these process conditions is illustrated in Figure 3. As expected, >90% process yield was achieved in all experimental runs. When flocculation was performed within the window of pH 6.0–7.0, 0.1–0.4% polymer dose and 10–40 mM stimulus, turbidity was reduced to <300 NTU after centrifugation. Further, the residual SmP E in the product stream was <20 ppm while HCP was decreased at least 1.6-fold.

## Clarification of Flocculated Cell Culture Using Clarisolve Depth Filters

We next examined the filtration performance of flocculated cell cultures. The capacity of Clarisolve 40MS and 60HX depth filters was first evaluated for SmP E treated cell cultures under conditions developed in the previous sections. The pressure drop threshold of 20 psi was never reached for treated Mab-A cell culture (Table 2). The control run achieved a 20 psi pressure drop across the Millistak+ D0HC/X0HC at a load of only 70 L/m^2^. In contrast, all Clarisolve filters demonstrated capacities of >250 L/m^2^ (Table 2). The filtrate turbidities were between 1 and 3 NTU.

**Table II:** Performance of depth and sterile filtration for SmP treated cultures

Flocculation conditions	mAb	Depth filter	Max Δ*P* (psi)	Capacity (L/m^2^)	Pool turbidity (NTU)	Sterile filter	Sterile filter capacity (L/m^2^)
Untreated	Mab-A	D0HC/X0HC	22.0	70	1.35	SHC	>10,000
0.1% SmP E, pH 7.0, 50 mM NaPi	Mab-A	40MS	11.8	261	1.19	SHF	>10,000
0.1% SmP E, pH 7.0, 50 mM NaPi	Mab-A	60HX	4.9	254	1.91	SHF	8,900
0.2% SmP E, pH 7.0, 50 mM NaPi	Mab-A	40MS	9.8	319	1.54	SHF	>10,000
0.2% SmP E, pH 7.0, 50 mM NaPi	Mab-A	60HX	10.1	329	1.22	SHF	>10,000
0.2% SmP E, pH 6.5, 25 mM NaPi	Mab-T	40MS	20.0	385	2.11	SHF	>10,000
0.2% SmP E, pH 6.5^1^, 25 mM NaPi	Mab-T	40MS	12.0	588	2.25	SHF	>10,000
0.4% SmP E, pH 7.0^1^, 10 mM NaPi	Mab-T	40MS	20.0	294	2.35	SHF	>10,000
0.2% SmP E, pH 6.5, 25 mM NaPi	Mab-T	60HX	20.0	327	2.96	SHF	>10,000
0.2% SmP E, pH 6.5^1^, 25 mM NaPi	Mab-T	60HX	20.0	310	3.32	SHF	>10,000
0.4% SmP E, pH 7.0^1^, 10 mM NaPi	Mab-T	60HX	20.0	310	3.25	SHF	>10,000

Untagged SmP E.

There was little difference on depth filtration performance in terms of filtrate turbidity for tagged and untagged SmP E treated cultures (Table 3). However, slightly higher Clarisolve 40MS filter capacity was demonstrated for the untagged polymer at 0.2% dose. Since the 40MS filter showed a higher or equivalent capacity compared with 60HX, it was chosen for the subsequent experiments (Table 3).

The sterilizing grade filter capacities for each filtrate from the primary clarification step (see Section Materials and Methods) are also shown in Table 2. D0HC/X0HC filtrate of the untreated culture, was tested on the Millipore Express SHC filter (0.5/0.2 µm PES membrane) while all other feeds were assessed on the Millipore Express SHF filter (0.2 µm PES membrane). *V*_max_ values in all runs except one were higher than 10,000 L/m^2^, indicating an efficient primary clarification process using the Clarisolve depth filter after the SmP E treatment.

## Process Integration/Small Scale Purification Production

We next integrated the developed flocculation and depth filtration steps into the mAb purification process, importantly incorporating a Protein A capture step, then evaluated the product quality in the Protein A eluate. Due to the limitation of available cell culture materials, purification runs were performed at a 1–2 L cell culture scale using 23 cm^2^ Clarisolve 40MS filter. At this scale, treated feed was sufficient to achieve filter loadings of >250 L/m^2^ (see Table 2). Differences in achieved throughputs on the Clarisolve 40 MS filter were not expected to impact product yield or impurity clearance. Further, scale-up of a flocculation process from liter-scale to >1,000 L has been successfully demonstrated previously (Kilander et al., 2007). Depth filters have also been shown to predictably perform at a process scale within 20% of the capacity achieved with bench-scale devices (Lutz et al., 2009). With proper controls, the SmP E clarification process should be amenable to large-scale mAb production.

Cell culture was treated with SmP E under several operating conditions utilized in the initial studies, in which 10 mM stimulus was explored as the worst-case scenario condition (Table 3). Due to the presence of extremely high level of aggregates in the cultures containing Mab-H or Mab-S (>37%), 0.4% SmP and 50 mM stimulus were used during the flocculation step. For Mab-T and Mab-A, untreated, clarified cell culture was used as the control. The flocculated cultures were clarified through 40MS depth filters in order to detect differences in process performance. As expected, acceptable process yield was achieved (Table 3). The residual HCP was reduced by 1.2- to 2.0-fold compared to the control, independent of flocculation conditions. In addition, >5 log_10_ of DNA clearance was achieved compared to the control, indicating superior DNA clearance as a result of the flocculation process. All these findings suggest that flocculation in the presence of SmP E leads to superior clarification process performance in terms of process yield and clearance of DNA and HCP impurities. Comparing the results in Table 3 with the previous flocculation experiments demonstrated similar yield, residual SmP E and impurity clearance performance from the milliliter to liter-scale.

**Table III:** Purification process performance with and without SmP E treatment

Flocculation					Depth filtration					Protein A chromatography			
mAb	pH	SMP E (%)	Stimulus (mM)	Filter	Yield (%)	HCP (FRV)	DNA (LRV)	SmP E (ppm)	Yield (%)	HCP (ppm)	DNA (pg/mg)	ProA (ng/mg)	HMW(%)
Mab-T	6.5	0.2^2010^	25	40MS	100	1.5	5.2	5.7	100	3.2	14.5	2.9	0.42
Mab-T	6.5	0.2	25	40MS	100	1.2	>6.1		100	3.1	1.8	1.7	0.35
Mab-T	7.0	0.4	10	40MS	100	1.3	>6.1		100	4.2	69.6	2.2	0.01
Mab-T	6.7	NA	NA	C0HC	100	NA	NA		98	212.9	1323.9	3.6	0.67
Mab-A	7.0	0.2^2010^	50	40MS	80	1.3	>6.20	14.0	83	17.9	ND	ND	0.73
Mab-A	7.0	NA	NA	C0HC	100	NA	NA		63	282.3	ND	ND	0.93
Mab-H	6.5	0.4	50	40MS	100	2.0	>6.4		100	< 1.0	6.4	1.6	0.03
Mab-S	6.5	0.4	50	40MS	100	1.5	6.3		98	6.0	1.5	2.0	0.24

NA, not applicable; ND, not determined; FRV, fold reduction value; LRV, log reduction value.

Tagged SmP E.

We also examined whether residual impurities were efficiently removed through the subsequent Protein A step (Table 3). The residual HCP in Protein A eluate was reduced to <20 ppm in all flocculation runs while it remained higher than 200 ppm in the control runs for both Mab-A and Mab-T. When untagged SmP E was used during flocculation under normal operating conditions, the residual DNA in Protein A eluate was <2 pg/mg-mAb. On the contrary, a higher level of residual DNA was observed under the worst-case scenario flocculating condition at which stimulus concentration was 10 mM (69.6 pg/mg), or using tagged SmP E (14.5 pg/mg). However residual DNA in all SmP E treated streams was significantly lower than that in the control (1323.9 pg/mg).

The level of mAb aggregates in the Protein A eluate was also evaluated by SE-HPLC. Small but consistent differences were observed in the presence and absence of SmP E. A lower level of HMW was achieved when flocculation was integrated into the purification process. A low level of leached Protein A was observed in all runs, which was in line with expectations for a typical Protein A chromatography manufacturing process. In summary, the SmP E flocculation process led to a better clarification process in terms of filter capacity and filtrate quality as well as robust reduction in residual HCP, host DNA, and HMW in the Protein A eluate. As such, this new flocculation process reduced the need for additional impurities removal steps in subsequent polishing chromatography.

## Residual SmP E Clearance

Due to potential toxicity purification processes employing flocculant polymers should be assessed for an acceptable residual polymer level, and an acceptable clearance level must be reached to ensure the safety of the drug product (Coffman and Shpritzer, 2010). Previous reports indicated that 1 ppm of cationic polymers showed little or no in vitro cytotoxicity (Fischer et al., 2003; Moghimi et al., 2005), suggesting that this level could be used as a reasonable target for acceptable residual polymer clearance.

**Figure 4:**
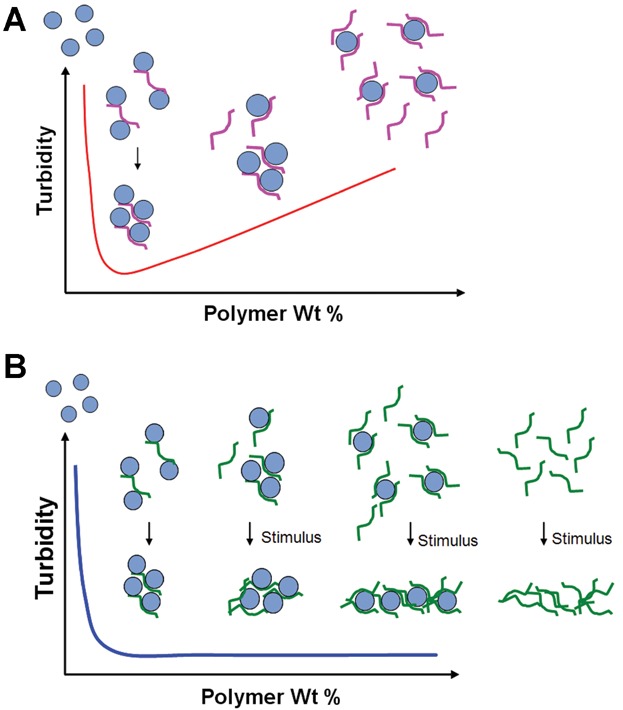
The hypothesis on the mechanism of action in flocculation processes using traditional flocculants (A) and SmP E (B).

With traditional polymeric flocculants, not only a narrow operating window on flocculant dose accompanied by inherent variation in cell culture makes development and manufacturing operations difficult but also free residual polymer molecules remaining in the culture after flocculation and clarification demand a strategy for monitoring and clearance (Fig. 4A). When SmP E is used, flocculated cells and cellular debris can be formed and be more efficiently removed by clarification through the introduction of a stimulus reagent which leads to the precipitation of the residual polymer (Fig. 4B). More importantly, excess SmP E is also subject to flocculation due to strong interactions between the polymer and stimulus molecules. Addition of stimulus therefore can extend the operating window on polymer dose. We have demonstrated that the residual SmP E was consistently removed to <20 ppm during the flocculation and clarification steps (Fig. 2 and Table 3).

We further examined the clearance of SmP E using Protein A chromatography. After the flocculated cell culture containing Mab-T was clarified, it was directly applied to a Protein A column at a loading capacity of 35 mg/mL-resin in a product capture mode. The residual SmP E in Protein A eluate was reduced to below the assay detection limit of 0.1 ppm.

## The Mechanism of HCP and HMW Clearance

More than 300 proteins were identified in supernatant from CHO cell cultures (Pezzini et al., 2011). These host cell proteins represent a diversity of proteins in molecular weight, isoelectric point (p*I*) and hydrophobicity. Approximately 50% of these proteins are acidic (p*I* < 6). 30% of proteins are neutral (p*I* 6–8). Fewer than 15% of proteins are basic (p*I* > 8). It is reasonable to believe that these proteins also possess hydrophobic patches or pockets. Here we evaluated SmP E precipitation as a potential alternative to the polishing chromatography processes for HCP reduction.

**Figure 5:**
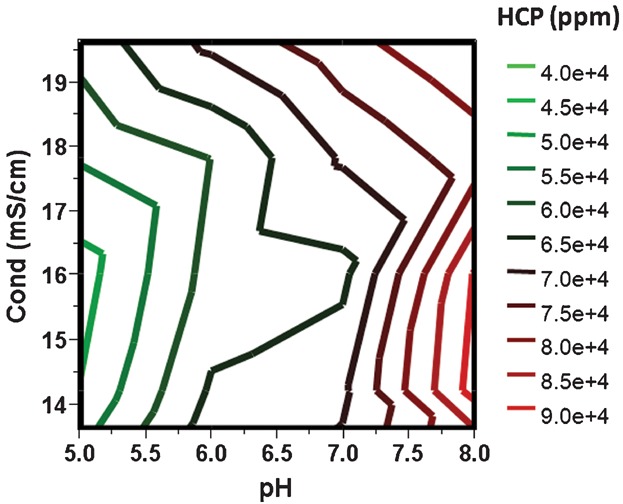
A contour plot analyzing the effect of cell culture pH and conductivity on HCP reduction in the Mab-T/SmP E flocculation process.

The HCP contour plot in Figure 2C(1) was adapted to reflect the impact of pH and conductivity (Fig. 5). The plot showed that decreasing flocculation pH from 8.0 to 5.0 progressively reduced the HCP level in product streams, while the impact of conductivity was marginal, strongly suggestive of a salt tolerant process. SmP E had a positive impact on HCP removal, as shown in Figure 2C(3). In the presence of SmP E, the optimal pH operating window for HCP reduction extended up to 8.0. In contrast, in the absence of SmP E, HCP reduction was marginal, suggesting that SmP E plays a major role in HCP clearance during this flocculation process. With the increase of flocculation pH from 5.0 to 8.0, the HCP level in the product streams increased, suggesting the ion exchange interactions alone could not explain the process behavior of HCP removal. In addition, absence of an optimal pH operating window in the neutral pH environment (pH 7.0–8.0) suggested that SmP E differs from traditional salt tolerant chromatography (Kang et al., 2012).

At pH 5.0, the lowest level of HCP was achieved, indicating that the pH neutralization of the host cell proteins may play a role in HCP removal. Since around 50% of host cell proteins are acidic (p*I* < 6) (Pezzini et al., 2011), a higher level of HCP removal is expected during this precipitation. Thus the pH neutralization of HCP alone could not explain less HCP reduction observed in the flocculation process.

Given that SmP E is also a hydrophobic polymer, we next examined the possible role of hydrophobic interactions between SmP E and host cell proteins. If the hydrophobic interactions contribute to HCP reduction, then those HCP that are hydrophobic in nature may be readily removed by the flocculation process. The efficient removal of these hydrophobic proteins during flocculation led to a high level of HCP clearance in the Protein A step. Thus residual HCP in the Protein A eluate reached the requirements of drug substance after the SmP E treatment. This is in agreement with a previous report on hydrophobic interactions between HCP and Protein A resin (Shukla and Hinckley, 2008). All these findings suggest a synergistic effect on HCP removal in SmP E flocculation process due to hydrophobic interaction, charge interaction, and precipitation.

The hydrophobic interaction mechanism can be further used to explain the removal of mAb aggregates (Table 3). Since mAb aggregates are more hydrophobic than monomers, hydrophobic interaction chromatography can be used in a product flow-through mode for aggregate removal (Yoo and Ghosh, 2012). In the SmP E flocculation process, aggregate removal was protein dependent. Particularly, when 0.4% SmP E was used, aggregates in Mab-H and Mab-T were reduced to <0.1%.

**Figure 6:**
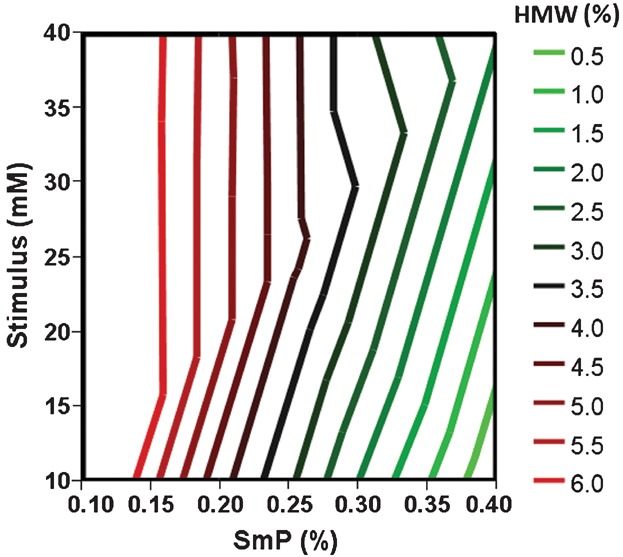
A contour plot analyzing the effect of SmP E dose and stimulus concentration on HMW reduction in the Mab-I/SmP E flocculation central composite design study.

We also examined whether aggregates can be efficiently reduced during the SmP E flocculation process when applied to a bispecific antibody, Mab-I, possessing a starting HMW level of 11.1%. A central composite design was used with SmP E from 0.1% to 0.4% and stimulus from 10 to 40 mM. The residual HMW contour plot is shown in Figure 6. Greater than 5.1% HMW reduction was observed for all conditions. When the SmP E dose was ≥0.25%, residual HMW after flocculation was reduced to <4.0%. This finding further suggests that the hydrophobic interaction plays an important role during SmP E flocculation process.

## Conclusion

The application of flocculation using a stimulus responsive polymer, SmP E, enables a robust alternative cell culture harvest step which may ameliorate bottlenecks in downstream processes, particularly for challenging monoclonal antibodies. In this study, a wide operating window of flocculation was established through full factorial experiments. Acceptable process yield and efficient clearance of HCP, host DNA, HMW, and residual SmP E were demonstrated using four model antibodies. More importantly, as a result of flocculation, HCP and host DNA were further removed in the Protein A step to levels that meet the requirements of drug substance and thus reduce the impurities load to subsequent purification steps. The mechanism of flocculation to enhance clarification of cells and cell debris and impurities clearance has been explored. A clearance study of residual SmP E was also performed. The applications of HCP and HMW removal through flocculation have been discussed. This novel and efficient harvest step can be easily integrated into current mAb production platforms.
