# Supplementary material for: Development of a novel and efficient cell culture flocculation process using a stimulus responsive polymer to streamline antibody purification processes
Source: Biotechnol Bioeng. 2013 Jun 29;110(11):2928–37. doi: 10.1002/bit.24969 (PMC3812681; doi:10.1002/bit.24969)
Supplement: Supplementary file 1 [file bit0110-2928-sd1.doc]

**S 1. Fluorescence labeling procedure for SmP and Poly(ethyleneimine)**

**SmP fluorescence labeling.** 150 ml of 10 % SmP in 1 M acetic acid solution was mixed with 1350 ml of Milli-Q® water in a 2 L glass jar till homogeneous. 1 M sodium hydroxide was added drop wise to the jar for a final pH of 8.5. Next, 35 mg of *N*-(4,4-Difluoro-1,3,5,7-Tetramethyl-4-Bora-3a,4a-Diaza-*s*-Indacene-2-yl) Iodoacetamide (BODIPY® 507/545 IA) (Life Technologies, Carlsbad, CA) was dissolved in 8 ml N,N-dimethylformamide (DMF) (Fisher Scientific, Waltham, MA) and was immediately added to the jar with continuous stirring. After a small amount of isopropanol (Thermo Fisher, New York, NY) was added to the jar and the solution was heated to 70°C and held for 1 hour with continuous stirring. The solution was allowed to cool to room temperature and continuously stirred for 3 days. The BODIPY 507/545 IA labeled SmP (SmP-BODIPY) was precipitated by drop wise addition of 2 M sodium phosphate until a final concentration of 100 mM sodium phosphate was reached. A red/orange solid was collected by decanting the supernatant and washing the precipitate with 50 mM sodium phosphate, pH 7.4. The collected solid was re-dissolved in 1 M acetic acid to result in a dark orange/red homogenous solution. The solution was concentrated using a 50 cm^2^ Biomax® polyethersulfone membrane with molecular weight cutoff (MWCO) of 5 kDa (EMD Millipore, Billerica, MA) and 10 volumes of diafiltration were performed to remove the free dye. The retentate was further concentrated using an Amicon® Stirred Cell model 8400, with a 1 kDa Ultracel® regenerated cellulose membrane (EMD Millipore, Billerica, MA) to a final concentration of 10% polymer, which was stored until used at 4^o^C in the dark. We have termed SmP-BODIPY as florescence tagged SmP or simply tagged SmP in this article.

**Poly(ethyleneimine) fluorescence labeling.** 15 g of poly(ethyleneimine) (PEI, average molecular weight of 60 kDa by gel permeation chromatography) (Sigma-Aldrich, St. Louis, MO) was mixed with 1 L of Milli-Q water in a 2-L glass jar till homogeneous. 1 M sodium hydroxide was added drop wise to the jar with stirring to a final pH of 9.0. 25 mg of BODIPY was dissolved in 6 ml DMF and was immediately added to the jar containing the PEI solution with continuous stirring. A small amount of isopropanol was added to the jar and the solution was heated to 70°C and held for 1 hour with continuous stirring. The solution was cooled to room temperature and stirred for 3 days. The solution was concentrated and diafiltrated as in SmP labeling process to a final concentration of 10 % (w/v) solids, which was stored until used at 4^o^C in the dark.
